# Tumor Long Interspersed Nucleotide Element-1 (LINE-1) Hypomethylation in Relation to Age of Colorectal Cancer Diagnosis and Prognosis

**DOI:** 10.3390/cancers13092016

**Published:** 2021-04-22

**Authors:** Naohiko Akimoto, Melissa Zhao, Tomotaka Ugai, Rong Zhong, Mai Chan Lau, Kenji Fujiyoshi, Junko Kishikawa, Koichiro Haruki, Kota Arima, Tyler S. Twombly, Xuehong Zhang, Edward L. Giovannucci, Kana Wu, Mingyang Song, Andrew T. Chan, Yin Cao, Jeffrey A. Meyerhardt, Kimmie Ng, Marios Giannakis, Juha P. Väyrynen, Jonathan A. Nowak, Shuji Ogino

**Affiliations:** 1 Program in MPE Molecular Pathological Epidemiology, Department of Pathology, Brigham and Women’s Hospital, Harvard Medical School, Boston, MA 02115, USA; nakimoto1@bwh.harvard.edu (N.A.); mzhao11@bwh.harvard.edu (M.Z.); tugai@bwh.harvard.edu (T.U.); zhongr@hust.edu.cn (R.Z.); mclau@bwh.harvard.edu (M.C.L.); fujiyoshi_kenji@med.kurume-u.ac.jp (K.F.); KISHIKAWAJ-SUR@h.u-tokyo.ac.jp (J.K.); haruki@jikei.ac.jp (K.H.); kouarima-gi@umin.ac.jp (K.A.); ttwombly@bwh.harvard.edu (T.S.T.); janowak@bwh.harvard.edu (J.A.N.); 2 Department of Gastroenterology, Nippon Medical School, Graduate School of Medicine, Tokyo 1138602, Japan; 3 Department of Epidemiology, Harvard T.H. Chan School of Public Health, Boston 02115, MA, USA; egiovann@hsph.harvard.edu (E.L.G.); hpkwu@channing.harvard.edu (K.W.); 4 Department of Epidemiology and Biostatistics, School of Public Health, Tongji Medical College, Huazhong University of Science and Technology, Wuhan 430030, China; 5 Channing Division of Network Medicine, Department of Medicine, Brigham and Women’s Hospital and Harvard Medical School, Boston, MA 02115, USA; poxue@channing.harvard.edu (X.Z.); achan@mgh.harvard.edu (A.T.C.); 6 Department of Nutrition, Harvard T.H. Chan School of Public Health, Boston, MA 02115, USA; mis911@mail.harvard.edu; 7 Clinical and Translational Epidemiology Unit, Massachusetts General Hospital and Harvard Medical School, Boston, MA 02114, USA; 8 Division of Gastroenterology, Massachusetts General Hospital, Boston, MA 02114, USA; 9 Department of Immunology and Infectious Diseases, Harvard T.H. Chan School of Public Health, Boston, MA 02115, USA; 10 Division of Public Health Sciences, Department of Surgery, Washington University in St. Louis, St. Louis, MO 63110, USA; yin.cao@wustl.edu; 11 Alvin J. Siteman Cancer Center, Washington University School of Medicine, St. Louis, MO 63110, USA; 12 Division of Gastroenterology, Department of Medicine, Washington University School of Medicine, St. Louis, MO 63110, USA; 13 Department of Medical Oncology, Dana-Farber Cancer Institute and Harvard Medical School, Boston, MA 02215, USA; jeffrey_meyerhardt@dfci.harvard.edu (J.A.M.); kimmie_ng@dfci.harvard.edu (K.N.); marios_giannakis@dfci.harvard.edu (M.G.); 14 Broad Institute of MIT and Harvard, Cambridge, MA 02142, USA; 15 Department of Medicine, Brigham and Women’s Hospital, Harvard Medical School, Boston, MA 02115, USA; 16 Cancer and Translational Medicine Research Unit, Medical Research Center Oulu, Oulu University Hospital, and University of Oulu, 90220 Oulu, Finland; 17 Cancer Immunology and Cancer Epidemiology Programs, Dana-Farber Harvard Cancer Center, Boston, MA 02215, USA

**Keywords:** carcinogenesis, colorectal neoplasms, epigenomics, genomic instability, long interspersed nuclear element, molecular pathology, retrotransposon, screening, transposable element, young-onset cancer

## Abstract

**Simple Summary:**

The rising incidence of early-onset cancers diagnosed before 50 years in many body sites (including the colorectum) is a growing concern. Despite the many studies of early-onset colorectal cancer, the characteristics of colorectal cancer diagnosed at age 50 or slightly after (close to age 50) have not been adequately examined. Although epigenetic alterations are considered to play a role in early-onset colorectal cancer, the association of LINE-1 hypomethylation (that reflects global DNA hypomethylation) with the age of colorectal cancer diagnosis has not been thoroughly clarified. Using a database of 1356 colorectal cancers, we found that tumor LINE-1 hypomethylation was increasingly more common with decreasing age of colorectal cancer diagnosis and was associated with higher colorectal cancer-specific mortality. Our findings support the “age continuum” model that has substantial implications in research on cancers in not only the colorectum but also many other body sites.

**Abstract:**

Evidence indicates the pathogenic role of epigenetic alterations in early-onset colorectal cancers diagnosed before age 50. However, features of colorectal cancers diagnosed at age 50–54 (hereafter referred to as “intermediate-onset”) remain less known. We hypothesized that tumor long interspersed nucleotide element-1 (LINE-1) hypomethylation might be increasingly more common with decreasing age of colorectal cancer diagnosis. In 1356 colorectal cancers, including 28 early-onset and 66 intermediate-onset cases, the tumor LINE-1 methylation level measured by bisulfite-PCR-pyrosequencing (scaled 0 to 100) showed a mean of 63.6 (standard deviation (SD) 10.1). The mean tumor LINE-1 methylation level decreased with decreasing age (mean 64.7 (SD 10.4) in age ≥70, 62.8 (SD 9.4) in age 55–69, 61.0 (SD 10.2) in age 50–54, and 58.9 (SD 12.0) in age <50; *p* < 0.0001). In linear regression analysis, the multivariable-adjusted β coefficient (95% confidence interval (CI)) (vs. age ≥70) was −1.38 (−2.47 to −0.30) for age 55–69, −2.82 (−5.29 to −0.34) for age 50–54, and −4.54 (−8.24 to −0.85) for age <50 (*P*_trend_ = 0.0003). Multivariable-adjusted hazard ratios (95% CI) for LINE-1 methylation levels of ≤45, 45–55, and 55–65 (vs. >65) were 2.33 (1.49–3.64), 1.39 (1.05–1.85), and 1.29 (1.02–1.63), respectively (*P*_trend_ = 0.0005). In conclusion, tumor LINE-1 hypomethylation is increasingly more common with decreasing age of colorectal cancer diagnosis, suggesting a role of global DNA hypomethylation in colorectal cancer arising in younger adults.

## 1. Introduction

The past few decades have witnessed a rising incidence of early-onset colorectal cancer, defined as colorectal cancer diagnosed before 50 years of age, in substantial parts of the world, particularly high-income countries [1,2,3]. The causes underlying this phenomenon remain unclear. The problem of the rising incidence of early-onset cancers in many body sites (including the colorectum) [4] has ranked as the top 2020 Provocative Question of the U.S. National Cancer Institute. Undoubtedly, there is a heightened interest in the biology and drivers of early-onset colorectal cancer [5,6].

Most of the previous studies regarding early-onset colorectal cancer adopted the dichotomy of age <50 vs. ≥50 years despite the lack of robust biological reasons to use age 50 as a cut point [7]. Only few studies examined tumor molecular features in relation to young age at onset, using models beyond the simple dichotomy at age 50 [8,9]. Therefore, the characteristics of colorectal cancer diagnosed at age 50 or after (but close to age 50) have not been adequately studied. For convenience, we hereafter designate colorectal cancer diagnosed at age 50–54 as “intermediate-onset colorectal cancer”.

Epidemiological evidence suggests associations between early-life exposures and colorectal cancer [10,11]. Exposures to certain exogenous (environmental) and endogenous factors may lead to cellular epigenetic alterations [12,13,14], which may play a role in tumor development in young age. Long interspersed nucleotide element-1 (LINE-1, also known as long interspersed nuclear element-1) constitutes approximately 17% of the human genome, and its methylation level is well correlated with the global DNA methylation status [15]. LINE-1 hypomethylation in colorectal cancer has been associated with younger age at diagnosis [16,17] and higher mortality [18,19,20]. However, LINE-1 hypomethylation of colorectal carcinoma in various age groups has not been thoroughly investigated. Our primary hypothesis was that tumor LINE-1 hypomethylation might be most common in early-onset colorectal cancer patients under 50, followed by those aged 50–54 (intermediate-onset), and least common in those aged ≥70. We also hypothesized that tumor LINE-1 hypomethylation might be associated with poor prognosis.

To test the primary hypothesis, we compared tumor LINE-1 methylation levels between age groups in a molecular pathological epidemiology database of 1356 colorectal cancer cases, including early-onset and intermediate-onset cases. We further assessed the relationship of age groups with tumor LINE-1 hypomethylation, controlling for lifestyle and clinical factors as well as other tumor molecular characteristics (microsatellite instability (MSI) status, CpG island methylator phenotype (CIMP), *KRAS* mutation, *BRAF* mutation, and *PIK3CA* mutation) in multivariate-adjusted linear regression analysis. In addition, we assessed the prognostic significance of the LINE-1 methylation level in multivariable Cox regression models.

## 2. Materials and Methods

### 2.1. Study Population

**Figure 1:**
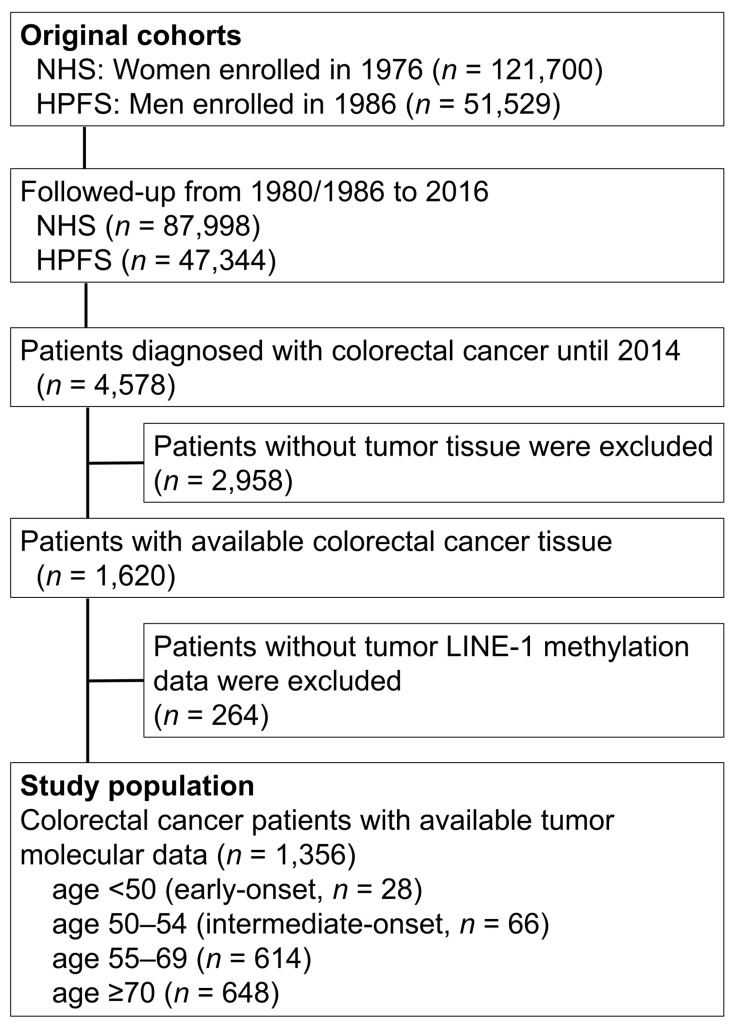
Flow chart of case selection in the Nurses’ Health Study and the Health Professionals Follow-up Study. Abbreviations: HPFS, Health Professionals Follow-up Study; LINE-1, long interspersed nucleotide element-1; NHS, Nurses’ Health Study.

We utilized two large prospective cohort studies in the United States, namely, the Nurses’ Health Study (with 121,700 women aged 30 to 55 years, followed up since 1976) and the Health Professionals Follow-up Study (with 51,529 men aged 40 to 75 years, followed up since 1986) [21] (Figure 1). In both cohorts, questionnaires were sent to participants to update information on their lifestyle factors and medical history, including diagnosis of colorectal cancer every two years. The response rate for each follow-up questionnaire was more than 90% for both cohorts. We used data on colorectal cancer family history, pack-years of smoking (using all available biennial questionnaires), and body mass index (using the latest available questionnaire before diagnosis). The National Death Index was used to identify unreported lethal cases of colorectal cancer. Participating physicians, who were blinded to exposure data, reviewed the medical records of identified colorectal carcinoma cases to confirm the disease diagnosis and to collect data on clinical characteristics (e.g., tumor size, tumor location, and the American Joint Committee on Cancer (AJCC) tumor, node, and metastases (TNM) classification). A single pathologist (S.O.) performed a centralized review of hematoxylin and eosin-stained tissue sections from all colorectal carcinoma cases blinded to other data [22]. Tumor differentiation was categorized as moderate or poor (>50% vs. ≤50% glandular area, respectively). As a result, we utilized a molecular pathological epidemiology database of 1356 colorectal cancer cases, which included 28 patients diagnosed before age 50 (early-onset cases) and 66 patients diagnosed at age 50–54 (herein referred to as “intermediate-onset” cases), with available tumor LINE-1 methylation data. On the basis of the colorectal continuum model, both colon and rectal cancers were included [23].

Informed consent was obtained from all study participants at enrollment. This study was approved by the institutional review boards of the Brigham and Women’s Hospital and Harvard T.H. Chan School of Public Health (Boston, MA, USA), and those of participating registries as required.

### 2.2. Assessment of LINE-1 Methylation Level

We collected formalin-fixed paraffin-embedded (FFPE) tissue blocks from hospitals where participants underwent tumor resection. Hematoxylin and eosin-stained slides of the tumors were reviewed, followed by marking of tumor areas. To enrich DNA from neoplastic cells, only tumor areas were macrodissected for DNA extraction. In a validation experiment for the LINE-1 assay [24], approximately 500 cancer cells from 5 anonymized cases were collected by laser capture microdissection (LCM) using an LCM instrument (MDS Analytical Technologies, CA, USA). Specimens were suspended in 140 μL of tissue lysate solution (pH 8, 1 mg/mL proteinase K, 100 mmol/L Tris, pH 8, 10 mmol/L ethylenediaminetetraacetic acid, and 0.05 mg/mL tRNA) and incubated overnight at 50 °C. The lysate was aliquoted into seven tubes (each containing 18 μL of tissue lysate) and stored at −20 °C till sodium bisulfite modification was performed.

The treatment of tissue lysates was conducted as previously reported [17,24]. Finally, we used the eluted DNA (80 μL volume) for pyrosequencing and MethyLight analysis [17,24].

**Figure 2:**
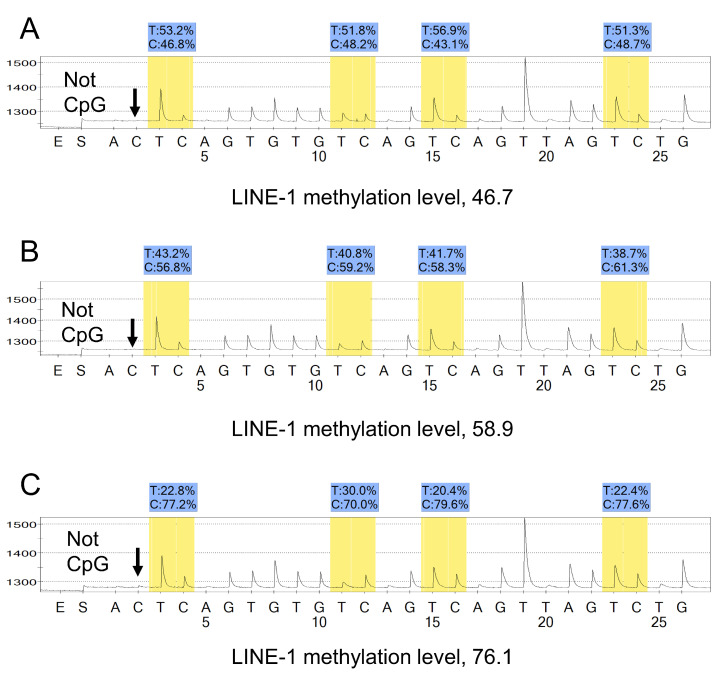
Pyrosequencing assay to measure LINE-1 methylation. (**A**) Tumor with LINE-1 hypomethylation (methylation level of 46.7). (**B**) Tumor with LINE-1 methylation level of 58.9. (**C**) Tumor with LINE-1 methylation level of 76.1. In panels A–C, the % numbers (in blue shade) are proportions of T and C nucleotides at each CpG site after bisulfite conversion. The proportion of C nucleotides (%) can be interpreted as the methylation level of each CpG site. The first, third, and fourth CpG sites follow mononucleotide T repeats, resulting in higher T peaks (in yellow shade) than the second CpG site. Accordingly, the proportion of C nucleotides (%) has been adjusted. No residual C nucleotides at the non-CpG site are indicated by the arrows, providing evidence for a complete bisulfite conversion reaction. We used the average of the proportions of C nucleotides at the 4 CpG sites as the LINE-1 methylation level (a scale of 0 to 100) of each tumor. To avoid confusion with other % numbers, “%” is not used for this LINE-1 methylation level to keep consistency between the text, tables, and figures. Abbreviation: LINE-1, long interspersed nucleotide element-1.

We performed the polymerase chain reaction (PCR) and pyrosequencing assay using the PyroMark kit (No. 978703, Qiagen, Valencia, CA, USA). This assay was conducted to amplify a region of the LINE-1 element (position 305 to 331 in accession No. X58075) which includes 4 CpG sites (Figure 2). The PCR condition was as follows; (1) 95 °C for 20 s (45 cycles); (2) 50 °C for 20 s and 72 °C for 20 s; and (3) 72 °C for 5 min. Using the Pyrosequencing Vacuum Prep Tool (Qiagen), we purified the biotinylated PCR product to make single-stranded nucleotides as a template in a pyrosequencing reaction. Then, we performed pyrosequencing reactions in the PSQ HS 96 System (Qiagen). The nucleotide dispensation order was as follows: 5′-ACT CAG TGT GTC AGT CAG TTA GTC TG-3′. We examined a single cytosine at a non-CpG site within PCR products to ensure successful bisulfite conversion of unmethylated cytosine based on evidence that the non-CpG cytosine in LINE-1 repetitive sequences was rarely methylated [25].

**Figure 3:**
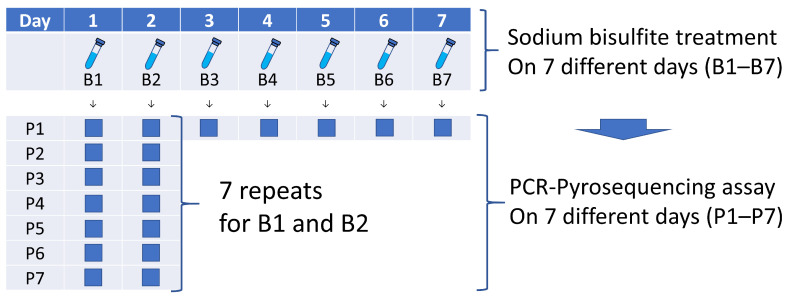
Validation procedure to assess precision of bisulfite conversion and PCR-pyrosequencing. Bisulfite conversion was performed on seven aliquots (B1 to B7) from each specimen. PCR-pyrosequencing was performed for seven bisulfite-treated specimens (B1 to B7) and was repeated seven times on two specimens (B1 and B2) on seven different days (P1 to P7). Abbreviation: PCR, polymerase chain reaction.

We calculated the percentage of the amount of C nucleotides divided by the sum of the amounts of C and T nucleotides at each CpG site. We calculated the average of the relative amounts of C nucleotides in the 4 CpG sites in LINE-1. This average percentage value (a unitless number on a scale of 0 to 100) was used as the LINE-1 methylation level of each tumor. To avoid confusion with other % numbers, we did not use “%” in this measure of the LINE-1 methylation level. Figure 3 shows the validation procedure to assess the precision of bisulfite conversion and PCR-pyrosequencing. We performed PCR-pyrosequencing seven times on each bisulfite-treated DNA and ensured a high precision of the LINE-1 methylation pyrosequencing assay [24]. We previously showed that DNA hypomethylation could be measured by using manual dissection without an LCM, and that the precision of measurement by using manual dissection was superior to cancer cells collected by LCM [24].

### 2.3. Assessments of Other Tumor Characteristics

DNA was extracted from archival FFPE blocks of normal and carcinomatous colorectal tissue. Methylation status of eight CIMP-specific promoters (*CACNA1G*, *CDKN2A*, *CRABP1*, *IGF2*, *MLH1*, *NEUROG1*, *RUNX3*, and *SOCS1*) was defined by the MethyLight assay using bisulphite-treated DNA, as described previously [19,23,26] (following nomenclature recommendations for genes and products by an expert panel [27]). CIMP-high was defined as ≥6/8 methylated markers using the 8-marker CIMP panel, CIMP-low as 1–5/8 methylated promoters, and CIMP-negative as 0/8 methylated promoters, according to previously established criteria [19,23]. Microsatellite instability (MSI) status was defined using PCR of 10 microsatellite markers (D2S123, D5S346, D17S250, BAT25, BAT26, BAT40, D18S55, D18S56, D18S67, and D18S487); MSI-high was defined as the presence of instability in ≥30% of the markers, as previously described [23,26]. PCR and pyrosequencing were performed for *KRAS* (codons 12, 13, 61, and 146), *BRAF* (codon 600), and *PIK3CA* (exons 9 and 20) [23,28].

### 2.4. Statistical Analyses

All statistical analyses were performed using the SAS software (version 9.4, SAS Institute, Cary, NC, U.S.). All *p* values were two-sided. We used the stringent two-sided α level of 0.005, as recommended by a panel of expert statisticians [29]. Our primary hypothesis testing was an assessment of the association of age at diagnosis (age groups) with LINE-1 methylation level. All other assessments were secondary analyses. Spearman’s correlation test was used to examine the association between four age groups and categorical data (or continuous values of LINE-1 methylation level). To control for other factors, multivariable-adjusted linear regression analyses were conducted with LINE-1 methylation level as an outcome variable. We initially included the following covariates: sex, body mass index (<30 vs. ≥30 kg/m^2^), pack-years of smoking (0 vs. 1–39 vs. ≥40), family history of colorectal cancer in any first-degree relative (present vs. absent), tumor location (proximal colon vs. distal colon vs. rectum), CIMP status (CIMP-negative/low vs. CIMP-high), MSI status (non-MSI-high vs. MSI-high), *KRAS* mutation (mutant vs. wild-type), *BRAF* mutation (mutant vs. wild-type), and *PIK3CA* mutation (mutant vs. wild-type). A backward elimination was conducted with a threshold *p* value of 0.05 to select variables for the final model. Cases with missing data (body mass index (0.4%), pack-years of smoking (4.4%), family history of colorectal cancer (0.7%), tumor location (0.5%), MSI status (2.8%), CIMP status (4.9%), *KRAS* mutation (6.4%), *BRAF* mutation (2.2%), and *PIK3CA* mutation (8.7%)) were imputed to the majority category of a given categorical covariate to limit the degrees of freedom of the models. Analyses using indicator variables for missing data in the variables in the final model yielded similar results (Table S1).

In survival analyses, cumulative survival probabilities were estimated with the Kaplan–Meier method, and a linear trend in survival probability across ordinal categories of LINE-1 methylation level was determined using the log-rank test for trend. Survival time was defined as the period from diagnosis of colorectal cancer to death or the end of follow-up, whichever came first. For the analyses of colorectal cancer-specific mortality, deaths due to other causes were censored. Multivariable Cox proportional hazard regression analyses were conducted for the colorectal cancer-specific survival according to the LINE-1 methylation level (≤45 vs. 45–55 vs. 55–65 vs. >65). A *p* value for trend was calculated using LINE-1 methylation level as a continuous variable with the same set of covariates. In the multivariable Cox regression model, we initially included the following covariates: age (continuous values), sex, body mass index (<30 vs. ≥30 kg/m^2^), pack-years of smoking (0 vs. 1–39 vs. ≥40), family history of colorectal cancer in any first-degree relative (present vs. absent), tumor location (proximal colon vs. distal colon vs. rectum), tumor differentiation (well to moderate vs. poor), AJCC disease stage (I-II vs. III-IV), CIMP status (CIMP-negative/low vs. CIMP-high), MSI status (non-MSI-high vs. MSI-high), *KRAS* mutation (mutant vs. wild-type), *BRAF* mutation (mutant vs. wild-type), and *PIK3CA* mutation (mutant vs. wild-type). A backward elimination was conducted with a threshold *p* of 0.05 to select variables for the final models. Cases with missing data (body mass index (0.4%), pack-years of smoking (4.4%), family history of colorectal cancer (0.7%), tumor location (0.5%), tumor differentiation (0.6%), AJCC disease stage (9.1%), MSI status (2.8%), CIMP status (4.9%), *KRAS* mutation (6.4%), *BRAF* mutation (2.2%), and *PIK3CA* mutation (8.7%)) were imputed to the majority category of a given categorical covariate to limit the degrees of freedom of the models. The proportionality of the hazard assumption was assessed using a time-varying covariate, which is an interaction term of survival time and LINE-1 methylation level. The proportionality of the hazard assumption was satisfied for the analyses of cancer-specific survival (*p* = 0.34).

### 2.5. Use of Standardized Official Symbols

We use HUGO (Human Genome Organisation)-approved official symbols for genes and gene products, including BRAF, CACNA1G, CDKN2A, CRABP1, IGF2, KRAS, MLH1, NEUROG1, PIK3CA, RUNX3, and SOCS1, all of which are described at www.genenames.org accessed on 20 April 2021. The official symbols are italicized to differentiate from non-italicized colloquial names that are used along with the official symbols. 

## 3. Results

In this study, we utilized a database of 1356 colorectal cancer cases with available tumor LINE-1 methylation data in the two prospective cohort studies, including 28 early-onset cases (age <50) and 66 intermediate-onset cases (age 50–54). Tumor LINE-1 methylation levels (unitless values on a scale of 0 to 100; derived from percentage numbers) ranged from 23.1 to 93.8 (mean 63.6; standard deviation (SD) 10.1).

**Figure 4:**
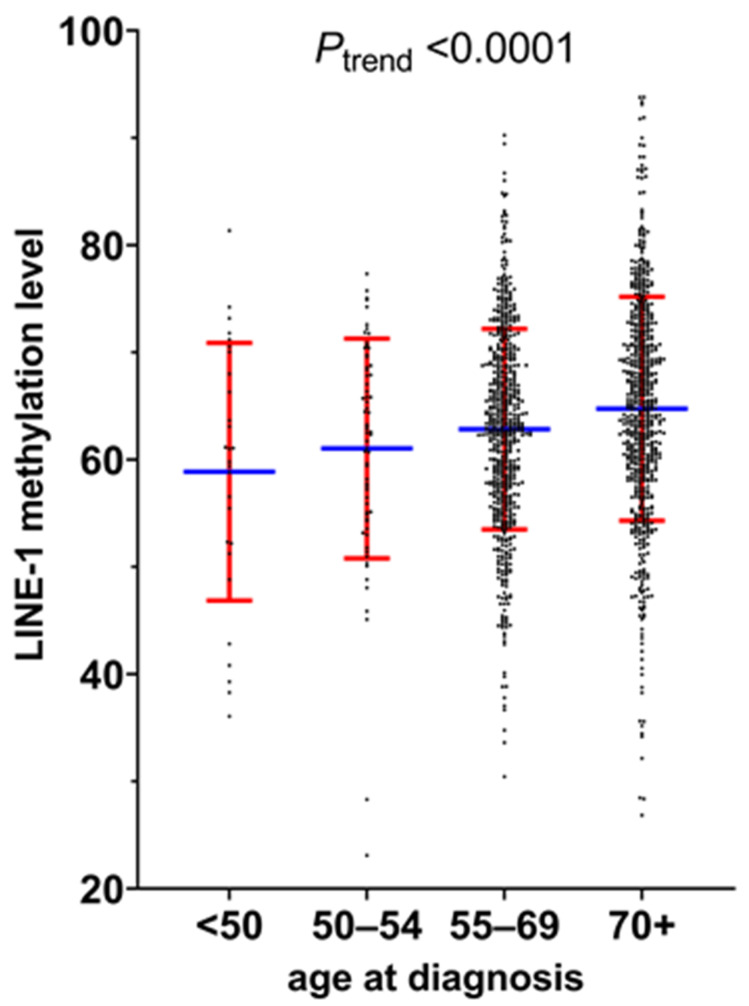
Distribution of tumor LINE-1 methylation levels in different age groups. In the scatter dot plot, the blue horizontal bar marks the mean and the red horizontal bar indicates the standard deviation of tumor LINE-1 methylation levels in each age group. A significant difference was observed between four age groups (≥70, 55–69, 50–54, and <50) (*P*_trend_ <0.0001). Abbreviation: LINE-1, long interspersed nucleotide element-1.

Table 1 summarizes the clinical, pathological, and molecular characteristics according to four age groups of ≥70, 55–69, 50–54, and <50 years (Table S2 showing data using further categorization of ≥70, 65–69, 60–64, 55–59, 50–54, and <50 years). In our primary hypothesis testing, younger age at diagnosis was associated with a lower tumor LINE-1 methylation level (*p* < 0.0001). The mean (with standard deviation (SD)) of the LINE-1 methylation level was 64.7 (SD 10.4) in age ≥70, 62.8 (SD 9.4) in age 55–69, 61.0 (SD 10.2) in age 50–54, and 58.9 (SD 12.0) in age <50 (*p* < 0.0001) (Figure 4).

We categorized the LINE-1 methylation level into four ordinal subtypes using arbitrary cut points of 45, 55, and 65 (Table 1). A tumor LINE-1 methylation level of ≤55 was uncommon in colorectal cancer patients aged >70 and those aged 55–69, but more common in those aged 50–54 and most common in those aged <50 (*p* = 0.0005). Conversely, a tumor LINE-1 methylation level of >65 was most common in patients aged >70, followed by those aged 55–69 and those aged 50–54, and least common in those aged <50.

To adjust for other factors, we conducted multivariable-adjusted linear regression analysis that could assess the association of age at diagnosis with the tumor LINE-1 methylation level as a continuous variable (Table 2). As shown in Table 1, early-onset colorectal cancers in this dataset were associated with some clinical and tumor molecular features, such as female sex, rectal location, non-MSI-high status, and CIMP-negative status. There was evidence for a certain degree of confounding, manifested as a difference in the β coefficients (i.e., difference in the mean LINE-1 methylation level by a given variable) between the unadjusted and adjusted models. However, even after the adjustment in the multivariable model, there was a highly significant association of four age groups with the LINE-1 methylation level (*P*_trend_ < 0.0001 across the age groups). Compared to patients aged ≥70, the multivariable-adjusted β coefficient for the continuous LINE-1 methylation level was −1.38 (95% confidence interval (CI), −2.47 to −0.30) for age 55–69, −2.82 (95% CI, −5.29 to −0.34) for age 50–54, and −4.54 (95% CI, −8.24 to −0.85) for age <50 (*P*_trend_ = 0.0003 across the age groups) (Table 2).

**Figure 5:**
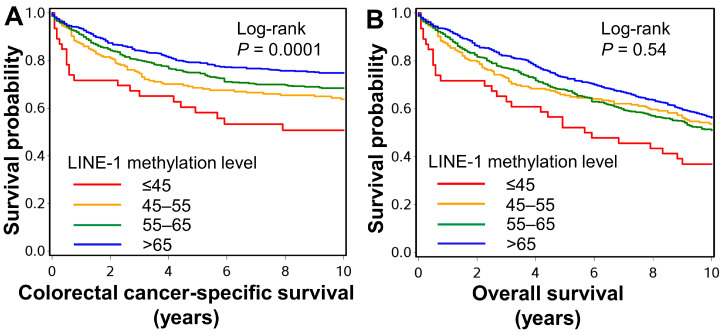
Kaplan–Meier survival curves of colorectal cancer-specific survival (**A**) and overall survival (**B**) according to the LINE-1 methylation level. The *p* values were calculated using the log-rank test for trend (two-sided).

In the survival analyses, using a dataset of 1352 cases with available survival data, we examined the prognostic impact of the LINE-1 methylation level. During the median follow-up time of 9.8 years (interquartile range, 3.8 to 16.2 years), 945 all-cause deaths, including 413 colorectal cancer-specific deaths, were observed. Kaplan–Meier analysis showed that LINE-1 hypomethylation was associated with higher colorectal cancer-specific mortality (log-rank *p* = 0.0001) (Figure 5 and Table 3). Multivariable Cox regression models indicated that LINE-1 hypomethylation was associated with higher colorectal cancer-specific mortality independent of tumor molecular features and patient characteristics. Multivariable-adjusted hazard ratios (95% CI) for LINE-1 methylation levels of ≤45, 45–55, and 55–65 (vs. >65) were 2.33 (1.49 to 3.64), 1.39 (1.05 to 1.85), and 1.29 (1.02 to 1.63), respectively (*P*_trend_ = 0.0005) (Table 4).

## 4. Discussion

We conducted this study to assess the association of age at colorectal cancer diagnosis with tumor LINE-1 hypomethylation, with a special focus on early-onset (age < 50) and intermediate-onset (age 50–54) cases. We leveraged the molecular pathological epidemiology database within the two prospective cohort studies that provided detailed information of clinicopathological features and tumor molecular profiles. Our findings of the association between early age of colorectal cancer diagnosis and tumor LINE-1 hypomethylation provide evidence for a greater pathogenic role of global DNA hypomethylation in colorectal cancers arising in younger age. Although the possible link between early-onset colorectal cancer and tumor LINE-1 hypomethylation has been reported [16], it has been unclear whether the link is independent of factors such as MSI status and CIMP. Furthermore, an open question is whether colorectal cancers diagnosed at age 50–54 (i.e., not early-onset cancer in the common definition, hence herein referred to as “intermediate-onset” cancer) have similar tumor characteristics to early-onset patients (or older patients). While replication is needed, our results suggest that intermediate-onset colorectal cancer patients (age 50–54) may exhibit tumor LINE-1 hypomethylation less commonly than early-onset cancer patients (age < 50) but more commonly than older patients. These findings do not suggest a sharp biological dichotomy of early-onset vs. later-onset colorectal cancer (with age 50 as a cut point), but rather support an “age continuum” model, which has recently been proposed [7].

The incidence of early-onset colorectal cancer has been increasing around the world [30]. Between 2000 and 2017, the age-adjusted annual incidence of colorectal cancer increased from 5.9 to 8.4 cases per 100,000 persons in the USA [31]. Accumulating evidence indicates that, compared to later-onset colorectal cancers (usually used for colorectal cancers diagnosed at age 50 or above), early-onset colorectal cancers are associated with rectal location, advanced stage at diagnosis, poor tumor differentiation, and signet ring cell histology [9,32,33,34,35,36]. Previous studies reported a relatively high prevalence of specific germline genetic features in young colorectal cancer patients [8,37,38]. Evidence indicates that the molecular characteristics of early-onset and later-onset colorectal cancers might differ [9,32,33,34,35,39]. Although certain early-life exposures, such as obesity, might be associated with early-onset colorectal cancer [11], the precise reason behind the increase in the incidence of early-onset colorectal cancer remains unclear, in part because most existing epidemiological studies lack precise early-life information [7]. A growing body of epidemiological evidence suggests that potential risk factors associated with early-onset colorectal cancer include male sex [40], family history of colorectal cancer [40,41], obesity [11,40], diet such as processed meat [40,41], Black and Asian ethnicities [40], and high intake of alcohol [41].

The genetic background of human populations is largely static over the short term. It is conceivable that environmental factors and their influence on epigenetics may play a role in the rise of early-onset colorectal cancer [7]. Evidence suggests that endogenous and exogenous exposures may act on cellular epigenetic modulators that regulate gene expression [12,42]. Hence, epigenetics is considered to serve as a bridge between the environment and phenotypes [7,14]. Hence, there is importance in investigating the association between age of onset and epigenetic alterations in cancer [16,17]. Aberrant epigenetic processes, which may be promoted by diet, lifestyle, and environmental exposures throughout the life course, likely underlie the increasing incidence of various cancer types, which were observed more commonly in old individuals in young adults.

DNA methylation is an essential epigenetic process that modulates gene expression. Global DNA hypomethylation is likely a manifestation of widespread epigenomic instability that plays a role in carcinogenesis [15]. A recent study suggested that LINE-1 retrotransposition (which may be caused by LINE-1 hypomethylation) may be a major mechanism that causes somatic structural variation in various tumor types including colorectal cancer [43]. Previous studies have reported that tumor LINE-1 hypomethylation, which has been correlated with global DNA hypomethylation [44,45], is associated with younger age of onset [16,17], higher T stage [46], and shorter survival [16,19,20,46,47,48,49,50] in colorectal cancer. The tumor LINE-1 methylation level in metastatic tumors appeared to be lower than that in primary tumors in colorectal cancer [51]. We found that LINE-1 hypomethylation was independently associated with non-MSI-high status and CIMP-negative/low status. The CIMP-high status is a major phenotype involved in colorectal cancer development, where CpG island methylation is a mechanism of silencing gene expression [52,53]. We have previously reported that CIMP-high tumors are associated with proximal colon location, female sex, poor differentiation, MSI-high status, and *BRAF* mutations [54]. To our knowledge, the current study is the largest to evaluate tumor LINE-1 methylation in different age groups, including early-onset and intermediate-onset colorectal cancers.

In this study, we examined whether the prevalence of LINE-1 hypomethylation abruptly varied at age 50 or gradually changed according to age. Although age 50 is usually defined as the cut point for early-onset vs. later-onset colorectal cancer, our current study provides no evidence that the molecular characteristics abruptly change at the age of 50. Rather, our data provide support for the “age continuum” model [7], with regard to tumor LINE-1 hypomethylation, which reflects global DNA hypomethylation. This age continuum model in colorectal cancer has important biological and clinical implications. It is possible that the recommended starting age of 50 for colorectal cancer screening in the past provided the ready rationale of the dichotomy model (with the cut point at age 50) in colorectal cancer research. The past screening practice might have influenced the incidence of colorectal cancer around age 50 in complex ways. Recently, the American Cancer Society [55], the U.S. Preventive Services Task Force, and the American College of Gastroenterology [56] recommended starting screening at the age of 45 instead of 50; therefore, monitoring how colorectal cancer incidence will change accordingly is an important element of early-onset colorectal cancer research moving forward. Our current study highlights the importance of considering the plausible “age continuum” model in research on cancers in not only the colorectum but also many other body sites.

Our study has limitations. First, the sample size of patients with early-onset colorectal cancer analyzed in this study was limited. Nonetheless, a moderate number of intermediate-onset cases and a large number of older patients in the molecular pathological epidemiology database enabled us to detect the statistically significant trend across the ordinal age groups of colorectal cancer. Second, there existed unmeasured and/or residual confounding in this observational study. However, we attempted to adjust for a number of factors such as body mass index, cigarette smoking, colorectal cancer family history, tumor location, CIMP, and MSI status in our multivariable-adjusted linear regression analysis. Third, most of the subjects in this study were non-Hispanic Whites. Therefore, a replication of findings in other populations is needed. Fourth, the bisulfite sequencing could not differentiate 5-methylcytosine from 5-hydroxymethylcytosine. Therefore, there was a possibility that some of the methylated CpG sites identified in the study might be due to 5-hydroxymethylcytosines.

The primary strength of this study was the utilization of the molecular pathological epidemiology approach [57,58,59,60,61,62,63], together with a large database of colorectal carcinoma cases, which integrated epidemiological, clinical, pathological, and tumor molecular features. This comprehensive database enabled us to conduct multivariable analysis adjusting for an extensive group of covariates. Moreover, the study subjects of incident colorectal cancer cases in the two prospective cohort studies were derived from over 100 hospitals throughout the U.S., which increases the generalizability of our findings, compared to studies based on a limited number of hospitals. In addition, our LINE-1 methylation assay on archival colorectal tumor tissue was extensively validated with well-documented robust performance characteristics including high precision [64].

## 5. Conclusions

This study indicates that younger age of colorectal cancer diagnosis is associated with lower LINE-1 methylation levels, potentially reflecting a greater pathogenic role of global DNA hypomethylation in colorectal cancer arising in younger adults. These findings support the “age continuum” model (as opposed to the common age dichotomy model at 50 years) in terms of tumor LINE-1 hypomethylation. The proposed “age continuum” model has substantial implications in research on cancers in the colorectum as well as other body sites.

## Figures and Tables

**Table 1:** Clinical, pathological, and molecular characteristics of colorectal cancer cases according to age at diagnosis.

Characteristics ^a^	Total No. (*n* = 1356)	Age at Diagnosis				*p* Value ^b^
		<50	50–54	55–69	≥70	
		(*n* = 28)	(*n* = 66)	(*n* = 614)	(*n* = 648)	
Sex						<0.0001
Female (NHS)	742 (55%)	21 (75%)	46 (70%)	378 (62%)	297 (46%)	
Male (HPFS)	614 (45%)	7 (25%)	20 (30%)	236 (38%)	351 (54%)	
Body mass index						0.42
<30 kg/m^2^	1099 (81%)	23 (82%)	54 (82%)	491 (80%)	531 (82%)	
≥30 kg/m^2^	251 (19%)	5 (18%)	12 (18%)	121 (20%)	113 (18%)	
Pack-years of smoking						0.020
0	531 (41%)	14 (50%)	40 (63%)	233 (39%)	244 (40%)	
1–39	547 (42%)	13 (46%)	24 (37%)	257 (43%)	253 (41%)	
≥40	219 (17%)	1 (3.6%)	0	103 (17%)	115 (19%)	
Family history of colorectal cancer						0.75
Absent	1077 (80%)	22 (79%)	51 (80%)	492 (81%)	512 (80%)	
Present	270 (20%)	6 (21%)	13 (20%)	119 (19%)	132 (20%)	
Tumor location						<0.0001
Proximal colon	641 (48%)	6 (21%)	26 (39%)	258 (42%)	351 (54%)	
Distal colon	410 (30%)	14 (50%)	26 (39%)	204 (33%)	166 (26%)	
Rectum	298 (22%)	8 (29%)	14 (22%)	150 (25%)	126 (20%)	
pT stage						0.76
pT1	143 (12%)	3 (11%)	4 (6.4%)	76 (13%)	60 (10%)	
pT2	259 (21%)	8 (30%)	13 (21%)	105 (19%)	133 (23%)	
pT3	773 (62%)	13 (48%)	41 (65%)	357 (63%)	362 (62%)	
pT4	67 (5.4%)	3 (11%)	5 (8.0%)	28 (5.0%)	31 (5.3%)	
pN stage						0.048
pN0 (0)	758 (63%)	16 (62%)	25 (45%)	346 (63%)	371 (65%)	
pN1 (1–3)	277 (23%)	4 (15%)	18 (33%)	127 (23%)	128 (23%)	
pN2 (≥4)	166 (14%)	6 (23%)	12 (22%)	77 (14%)	71 (12%)	
AJCC disease stage						0.026
I	315 (26%)	9 (32%)	9 (15%)	141 (25%)	156 (27%)	
II	394 (32%)	7 (25%)	13 (22%)	180 (32%)	194 (34%)	
III	351 (28%)	9 (32%)	26 (43%)	162 (29%)	154 (26%)	
IV	172 (14%)	3 (11%)	12 (20%)	83 (14%)	74 (13%)	
Tumor differentiation						0.59
Well to moderate	1212 (90%)	26 (93%)	59 (89%)	551 (90%)	576 (89%)	
Poor	136 (10%)	2 (7.0%)	7 (11%)	59 (9.7%)	68 (11%)	
LINE-1 methylation level						<0.0001
(0 to 100 (percent) scale), mean ± SD	63.6 ± 10.1	58.9 ± 12.0	61.0 ± 10.2	62.8 ± 9.4	64.7 ± 10.4	
LINE-1 methylation level						0.0005
≤45	47 (3.5%)	5 (18%)	2 (3.0%)	21 (3.4%)	19 (2.9%)	
45–55	206 (15%)	4 (14%)	15 (23%)	98 (16%)	89 (14%)	
55–65	499 (37%)	10 (36%)	23 (35%)	245 (40%)	221 (34%)	
>65	604 (45%)	9 (32%)	26 (39%)	250 (41%)	319 (49%)	
MSI status						<0.0001
Non-MSI-high	1101 (84%)	26 (100%)	56 (89%)	520 (87%)	499 (79%)	
MSI-high	217 (16%)	0 (0%)	7 (11%)	80 (13%)	130 (21%)	
CIMP status						<0.0001
Negative	553 (43%)	17 (61%)	37 (58%)	270 (46%)	229 (38%)	
Low	507 (39%)	10 (36%)	23 (36%)	240 (40%)	234 (39%)	
High	230 (18%)	1 (3.6%)	4 (6.3%)	83 (14%)	142 (23%)	
*KRAS* mutation						0.22
Wild-type	737 (58%)	16 (62%)	41 (66%)	346 (59%)	334 (57%)	
Mutant	532 (42%)	10 (38%)	21 (34%)	245 (41%)	256 (43%)	
*BRAF* mutation						0.024
Wild-type	1124 (85%)	24 (92%)	56 (89%)	522 (86%)	522 (83%)	
Mutant	202 (15%)	2 (7.7%)	7 (11%)	83 (14%)	110 (17%)	
*PIK3CA* mutation						0.63
Wild-type	1039 (84%)	22 (85%)	49 (86%)	463 (84%)	505 (83%)	
Mutant	199 (16%)	4 (15%)	8 (14%)	87 (16%)	100 (17%)	

^a^ Percentage indicates the proportion of patients with a specific clinical, pathological, or molecular characteristic among all patients or in the strata of age at diagnosis. ^b^ Spearman’s correlation test was used to examine the association between four age groups (≥70, 55–69, 50–54, and <50) and categorical data (or continuous value of LINE-1 methylation level). Abbreviations: AJCC, American Joint Committee on Cancer; CIMP, CpG island methylator phenotype; HPFS, Health Professionals Follow-up Study; LINE-1, long interspersed nucleotide element-1; MSI, microsatellite instability; NHS, Nurses’ Health Study; SD, standard deviation.

**Table 2:** Linear regression analysis to predict LINE-1 methylation level (outcome) by four age groups (predictor).

Variables in the Final Model	β Coefficient ^a^ (Change in Mean LINE-1 Methylation Levels by a Given Variable)			
	Univariable (Unadjusted) (95% CI)	*p* Value	Multivariable-Adjusted ^b^ (95% CI)	*p* Value
Age at diagnosis		<0.0001 ^c^		0.0003 ^c^
<50	−5.87 (−9.65 to −2.09)		−4.54 (−8.24 to −0.85)	
50–54	−3.70 (−6.23 to −1.17)		−2.82 (−5.29 to −0.34)	
55–69	−1.90 (−3.00 to −0.80)		−1.38 (−2.47 to −0.30)	
≥70	Referent		Referent	
Family history of colorectal cancer		0.13		0.029
Absent	Referent		Referent	
Present	−1.03 (−2.37 to 0.31)		−1.45 (−2.75 to −0.15)	
MSI status		<0.0001		0.002
Non-MSI-high	Referent		Referent	
MSI-high	5.73 (4.30 to 7.16)		2.99 (1.14 to 4.84)	
CIMP status		<0.0001		<0.0001
Negative/low	Referent		Referent	
High	6.00 (4.61 to 7.39)		3.86 (2.05 to 5.67)	

^a^ The β coefficient represents a change (increase or decrease) in mean LINE-1 methylation level by a given variable. ^b^ The multivariable linear regression model initially included age, sex, body mass index, pack-years of smoking, family history of colorectal cancer in any first-degree relative, tumor location, MSI status, CIMP status, and *KRAS*, *BRAF*, and *PIK3CA* mutations. A backward elimination with a threshold *p* of 0.05 was performed to select variables in the final model. The variables listed in Table 2 remained in the final model. ^c^ *P*_trend_ was calculated by the linear trend across the ordinal age variable (≥70, 55–69, 50–54, and <50) in the linear regression model. Abbreviations: CI, confidential interval; CIMP, CpG island methylator phenotype; LINE-1, long interspersed nucleotide element-1; MSI, microsatellite instability.

**Table 3:** Number at risk of death during follow-up of patients according to tumor LINE-1 methylation levels

LINE-1 Methylation Level	Years					
	0	2	4	6	8	10
≤45	46	33	28	22	20	17
45–55	206	164	141	132	122	108
55–65	498	407	358	314	279	242
>65	602	519	468	420	365	296

This table shows the number of patients who remained alive and at risk of death at each time point after the diagnosis of colorectal cancer.

**Table 4:** Survival of colorectal cancer patients according to tumor LINE-1 methylation levels.

		Colorectal Cancer-Specific Survival			Overall Survival		
LINE-1 Methylation Level	No. of Cases	No. of Events	Univariable HR (95% CI)	Multivariable HR (95% CI) ^a^	No. of Events	Univariable HR (95% CI)	Multivariable HR (95% CI) ^a^
≤45	46	22	2.26 (1.41 to 3.62)	2.33 (1.49 to 3.64)	34	1.09 (0.70 to 1.69)	1.63 (1.07 to 2.49)
45–55	206	80	1.60 (1.22 to 1.16)	1.39 (1.05 to 1.85)	150	1.01 (0.84 to 1.23)	1.10 (0.90 to 1.33)
55–65	498	157	1.28 (1.03 to 1.60)	1.29 (1.02 to 1.63)	365	1.11 (0.96 to 1.27)	1.21 (1.05 to 1.40)
>65	602	154	Referent	Referent	396	Referent	Referent
*P*_trend_^b^			0.0002	0.0005		0.92	0.020

^a^ The multivariable Cox regression model initially included age, sex, body mass index, pack-years of smoking, family history of colorectal cancer in any first-degree relative, tumor location, tumor differentiation, disease stage, microsatellite instability, CpG island methylator phenotype, and *KRAS*, *BRAF*, and *PIK3CA* mutations. A backward elimination with a threshold *p* of 0.05 was used to select variables for the final models. ^b^ *P*_trend_ was calculated using LINE-1 methylation level as a continuous variable with the same set of covariates. Abbreviations: CI, confidence interval; HR, hazard ratio; LINE-1, long interspersed nucleotide element-1.

## Data Availability

The datasets generated and/or analyzed during the current study are not publicly available. Further information including the procedures to obtain and access data from the Nurses’ Health Studies and the Health Professionals Follow-up Study is described at https://www.nurseshealthstudy.org/researchers/ accessed on 20 April 2021 and https://sites.sph.harvard.edu/hpfs/for-collaborators/ accessed on 20 April 2021.

## Supplementary Materials

The following available online at https://www.mdpi.com/article/10.3390/cancers13092016/s1, Table S1: LINE-1 methylation level of colorectal cancer cases according to age at diagnosis, Table S2: Linear regression analysis to predict LINE-1 methylation level (outcome) by four age groups (predictor) using indicator variables for missing data.

## Abbreviations

AJCC	American Joint Committee on Cancer
CI	confidence interval
CIMP	CpG island methylator phenotype
FFPE	formalin-fixed paraffin-embedded
HR	hazard ratio
LCM	laser capture microdissection
LINE-1	long interspersed nucleotide element-1
MSI	microsatellite instability
PCR	polymerase chain reaction
SD	standard deviation
TNM	tumor, node, and metastases
