# Supplementary material for: Tumor Long Interspersed Nucleotide Element-1 (LINE-1) Hypomethylation in Relation to Age of Colorectal Cancer Diagnosis and Prognosis
Source: Cancers (Basel). 2021 Apr 22;13(9):2016. doi: 10.3390/cancers13092016 (PMC8122644; doi:10.3390/cancers13092016)
Supplement: Supplementary file 1 [file cancers-13-02016-s001.zip › cancers-1164965-supplementary.pdf]

Supplementary Materials: Tumor Long Interspersed Nucleotide Element-1 (LINE-1) Hypomethylation in Relation to Age of Colorectal Cancer Diagnosis and Prognosis

Naohiko Akimoto, Melissa Zhao, Tomotaka Ugai, Rong Zhong, Mai Chan Lau, Kenji Fujiyoshi, Junko Kishikawa, Koichiro Haruki, Kota Arima, Tyler S. Twombly, Xuehong Zhang, Edward L. Giovannucci, Kana Wu, Mingyang Song, Andrew T. Chan, Yin Cao, Jeffrey A. Meyerhardt, Kimmie Ng, Marios Giannakis, Juha P. Väyrynen, Jonathan A. Nowak and Shuji Ogino

Table S1. Linear regression analysis to predict LINE-1 methylation level (outcome) by four age groups (predictor) using indicator variables for missing data.

Variables in the model	β coefficient [a] (change in mean LINE-1 methylation levels by a given variable)			
	Univariable (unadjusted) (95% CI)	*P* value	Multivariable-adjusted [b] (95% CI)	*P* value
Age at diagnosis		< 0.0001 [c]		0.0002 [c]
<50	−5.87 (−9.65 to −2.09)		−4.79 (−8.49 to −1.10)	
50–54	−3.70 (−6.23 to −1.17)		−2.84 (−5.32 to −0.37)	
55–69	−1.90 (−3.00 to −0.80)		−1.38 (−2.47 to −0.30)	
≥70	Referent		Referent	
Family history of colorectal cancer		0.13		0.028
Absent	Referent		Referent	
Present	−1.03 (−2.37 to 0.31)		−1.45 (−2.75 to −0.15)	
MSI status		< 0.0001		0.0007
Non-MSI-high	Referent		Referent	
MSI-high	5.73 (4.30 to 7.16)		3.20 (1.35 to 5.06)	
CIMP status		<0.0001		<0.0001
Negative/low	Referent		Referent	
High	6.00 (4.61 to 7.39)		3.72 (1.90 to 5.55)	

[a] The β coefficient represents a change (increase or decrease) in mean LINE-1 methylation level by a given variable. [b] The multivariable linear regression model initially included age, family history of colorectal cancer in any first-degree relative, MSI status, and CIMP status. [c] P_{trend} was calculated by the linear trend across the ordinal age variable (≥70, 55–69, 50–54, and <50) in the linear regression model.-Abbreviations: CI, confidential interval; CIMP, CpG island methylator phenotype; LINE-1, long interspersed nucleotide element-1; MSI, microsatellite instability.

Table S2. LINE-1 methylation level of colorectal cancer cases according to age at diagnosis.

LINE-1 methylation level [0 to 100 (percent) scale]	Total No. (*n* = 1356)	Age at diagnosis						*P* value *
		<50 (*n* = 28)	50–54 (*n* = 66)	55–59 (*n* = 122)	60–64 (*n* = 191)	65–69 (*n* = 301)	≥70 (*n* = 648)	
								<0.0001
mean ± SD	63.6 ± 10.1	58.9 ± 12.0	61.0 ± 10.2	61.3 ± 10.3	61.7 ± 8.90	64.2 ± 9.07	64.7 ± 10.4	
								<0.0001
≤45	47 (3.5%)	5 (18%)	2 (3.0%)	8 (6.6%)	5 (2.6%)	8 (2.7%)	19 (2.9%)	
45–55	206 (15%)	4 (14%)	15 (23%)	23 (19%)	40 (21%)	35 (12%)	89 (14%)	
55–65	499 (37%)	10 (36%)	23 (35%)	41 (34%)	83 (43%)	121 (40%)	221 (34%)	
>65	604 (45%)	9 (32%)	26 (39%)	50 (41%)	63 (33%)	137 (45%)	319 (49%)	

* Spearman's correlation test was used to examine the association between six age groups (≥70, 65–69, 60–64, 55–60, 50–54, and <50) and categorical data (or continuous value) of LINE-1 methylation level. Abbreviations: LINE-1, long interspersed nucleotide element-1; SD, standard deviation.
